# First Discovery and Stucture-Activity Relationship Study of Phenanthroquinolizidines as Novel Antiviral Agents against *Tobacco Mosaic Virus* (TMV)

**DOI:** 10.1371/journal.pone.0052933

**Published:** 2012-12-28

**Authors:** Ziwen Wang, Anzheng Feng, Mingbo Cui, Yuxiu Liu, Lizhong Wang, Qingmin Wang

**Affiliations:** State Key Laboratory of Elemento-Organic Chemistry, Research Institute of Elemento-Organic Chemistry, Nankai University, Tianjin, China; Concordia University Wisconsin, United States of America

## Abstract

A series of phenanthroquinolizidine alkaloids **1**–**24** were prepared and first evaluated for their antiviral activity against *tobacco mosaic virus* (TMV). The bioassay results showed that most of these compounds exhibited good to excellent *in vivo* anti-TMV activity, of which compounds **1**, **2**, **15** and **16** displayed significantly higher activity than (*R*)-antofine and commercial Ningnanmycin at the same test condition. The substituents on the phenanthrene moiety play an important role for maintaining high *in vivo* antiviral activity. The introduction of 6-hydroxyl, which is proposed to interact with TMV RNA, did increased anti-TMV activity. The 14a*R*-configuration was confirmed to be the preferred antiviral configuration for phenanthroquinolizidine alkaloids. Introduction of hydroxy group at 15-position of phenanthroquinolizidine alkaloids increased activity for *S*-configuration but decreased activity for *R*-configuration. Present study provides fundamental support for development and optimization of phenanthroquinolizidine alkaloids as potential inhibitors of plant virus.

## Introduction

Plant viruses cause numerous diseases in a wide range of crop plant species and lead to an estimated $600 billion in annual losses worldwide [1]. *Tobacco mosaic virus* (TMV), the type member of Tobamovirus genus, is a prevalent plant pathogen all over the world and has the widest host range of over 885 plant species in 65 families. Most TMVs have infected a number of economically important crops and have induced general mosaic symptoms to cause significant economic losses worldwide [2]. It is found that in certain fields 90–100% of the plants show mosic or leaf necrosis by harvesting time. Therefore, this plant virus has the name “plant cancer” and is difficult to control.

**Figure 1:**
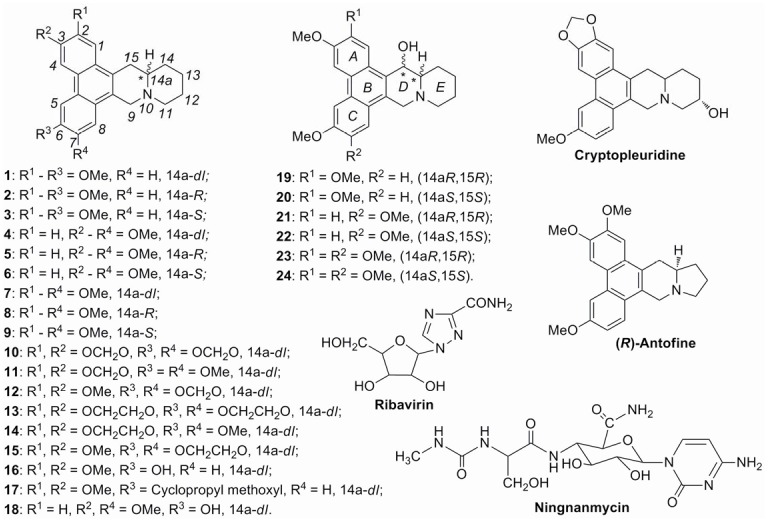
Chemical structures of compounds 1–24, Ribavirin, Ningnanmycin, Cryptopleuridine and (*R*)-Antofine.

To control viral plant diseases, many approaches have been used, including chemicals, breeding, cross-protection and transgenic plants. However, there are no control measures that can totally inhibit plant viruses after they have infected plants. Ningnanmycin (**Figure 1**), perhaps the most successful registered antiplant viral agent, displayed 56.0% *in vivo* curative effect at 500 µg/mL. Ribavirin (**Figure** **1**) is another widely used plantviral inhibitor, its inhibitory effects are also less than 50% at 500 µg/mL. Because of the unsatisfactory cure rate (30–60%) of common antiviral agents (Ningnanmycin, Ribavirin, Virus A, *et al*.) and economic loss of tobacco, many efforts have been done to develop novel, potent and structure concise antiviral agents. Some chemicals, such as triazolyl compounds [3], pyrazole derivatives [4], [5], cyanoacrylate derivatives [5], [6], α-aminophosphonate derivatives [5], [7], *N*-(pyrimidin-5-yl)-*N*′-phenylureas [8], and some natural products [9]–[11], have been found to possess antiviral activity. However, there are only a few reports on economically viable antiviral chemicals available for application in agriculture [12], so there still lies a great deal of scope for further research in this field.

Natural product-based agrochemicals offer advantages in that they can sometimes be specific to a target species and often have unique modes of action with little mammalian toxicity. Another benefit is their ability to decompose rapidly, thereby reducing their risk to the environment [13], [14]. An additional advantage is the natural products can be a candidate that possesses the desirable biological activities.

Phenanthroquinolizidine alkaloids are a small group of alkaloids existing in the *Lauraceae*, *Vitaceae* and *Urticaceae* family of plants. Only five of these alkaloids ((*R*)-cryptopleurine (**2**), (*R*)-boehmeriasin A (**5**), boehmeriasin B (**18**), cryptopleuridine and (14a*R*,15*R*)-15-hydroxycryptopleurine (**19**), **Figure 1**) have been isolated by now [15]–[18]. It has been reported that these alkaloids possess unique and interesting biological properties including vesicant [15], antimicrobial [19], antiviral [20], [21] and anti-tumor [22]–[24] activities. To date, most of the studies have been focused on anti-tumor activity in medicinal formulation. However, there have been no reports about the anti-TMV activity of phenanthroquinolizidine alkaloids in pesticide formulation.

In our previous work, the phenanthroindolizidine alkaloid (−)-antofine (**Figure 1**) was isolated from the aerial parts of *Cynanchum komarovii* and was first found to have good antiviral activity against TMV *in vitro* [25]. Further antiviral mechanism studies revealed that antofine has favorable interaction with origin of TMV RNA (oriRNA), likely exerting its virus inhibition by binding to oriRNA and interfering with virus assembly initiation [26]. Although phenanthroquinolizidine and phenanthroindolizidine are structure analogs, their biological properties are different [22]. Did the phenanthroquinolizidine alkaloids possess anti-TMV activity?

Based on the above findings, a series of phenanthroquinolizidine alkaloids **1**–**24** were prepared and systematically evaluated for their antiviral activity against TMV.

## Results and Discussion

### Chemistry

The alkaloids **1**–**9** [27], **18** [28], **19**–**24** [27] and acids **25a**–**f** [24] were prepared according to the corresponding reported procedures. Phenanthroquinolizidine alkaloids **10**–**17** were synthesized by using a practical synthetic approach involving coupling of the phenanthrene ring with 2-bromopyridine through a nucleophilic substitution reaction and subsequent reduction of the resulting pyridyl ketone as key steps.

**Figure 2:**
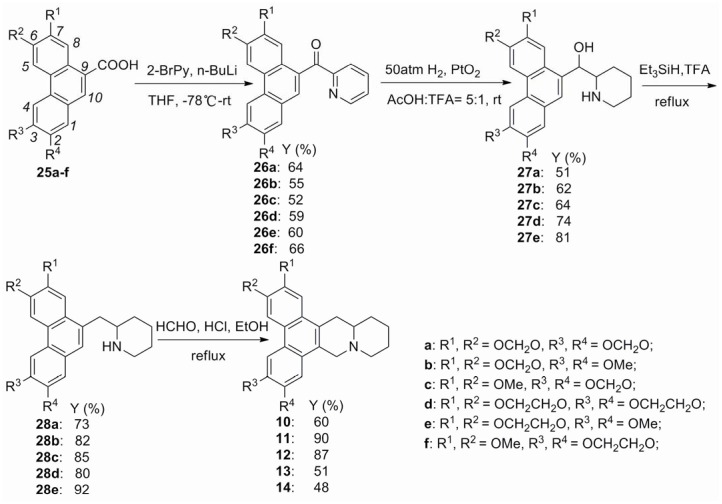
Synthesis of compounds 10–14.

As shown in **Figure 2**, 2-bromopyridine was treated with *n-*BuLi at –78°C to afford 2-lithiopyridine, which reacted smoothly with **25a**–**f** to give pyridyl ketones **26a**–**f**, respectively. By using PtO_2_ as the catalyst, the hydrogenation of **26a**–**e** proceeded smoothly in the solution of acetic acid and trifluoroacetic acid (v/v 5∶1) at 50 atm to give **27a**–**e**, respectively. It should be noted that pyridyl ketone **26f** could not been reduced under above reduction condition. The reduction of **27a**–**e** in Et_3_SiH/TFA at reflux afforded **28a**–**e**, which were then converted into phenanthroquinuolizidine alkaloids **10**–**14**, respectively, by using the Pictet–Spengler cyclomethylenation.

**Figure 3:**
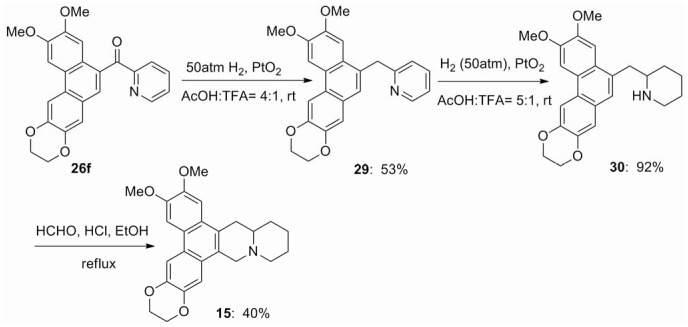
Synthesis of compound 15.

The hydrogenation of **26f** was carried out at several different conditions. The best result is that treatment of compound **26f** with PtO_2_ in the solution of acetic acid and trifluoroacetic acid (v/v 4∶1) at 50 atm gave the carbonyl group reduced intermediate **29** in 53% yield. Reduction of **29** using the similar procedure for the preparation of **27a**–**e** gave the key intermediate **30** in 92% yield, which was converted into phenanthroquinuolizidine alkaloid **15** by using the Pictet–Spengler cyclomethylenation (**Figure 3**).

**Figure 4:**
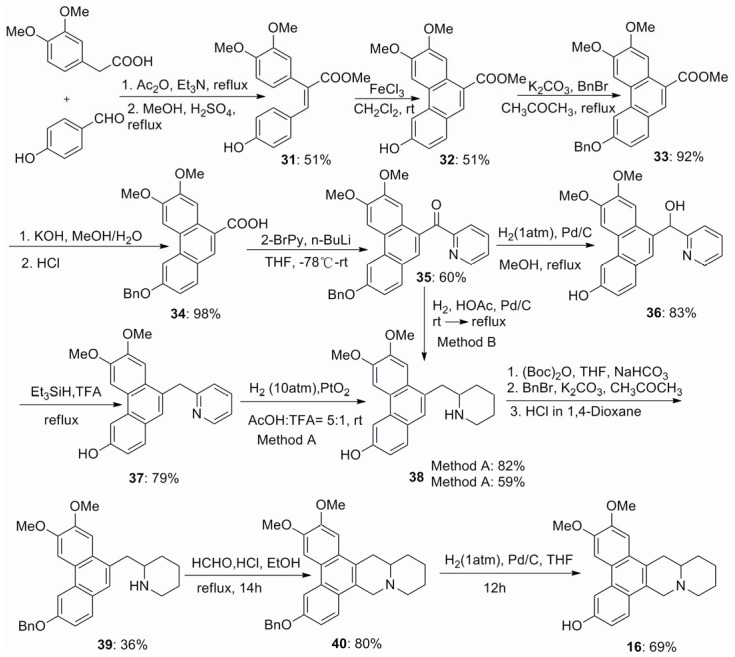
Synthesis of compound 16.

**Figure 5:**
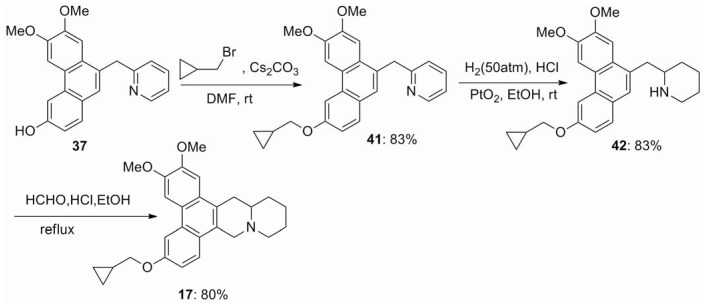
Synthesis of compound 17.

In order to further evaluate the effect of 6-hydroxy group on antiviral activity, which is proposed to interact with TMV RNA [26], phenanthroquinuolizidine alkaloids **16** and **17** were designed and synthesized. Phenanthrene-9-carboxylic acid **34** was synthesized by employing 2-(3,4-dimethoxyphenyl)acetic acid and 4-hydroxybenzaldehyde as the starting materials (**Figure 4**). Ester **33** was obtained by the conventional four steps previously reported in the literature [29]. Then hydrolysis of ester **33** gave acid **34**, which was treated with 2-lithiopyridine to afford the desired ketone **35**. The catalytic hydrogenation of **35** to give **38** was carried out under two different procedures. Treatment of compound **35** with Pd/C as the catalyst in reluxing methanol at 1 atm gave the alcohol **36** in 83% yield. Reduction of **36** using the Et_3_SiH/TFA system afforded the desired product **37**, which was further reduced using PtO_2_ as the catalyst in acetic acid and trifluoroacetic acid (v/v 5∶1) at 10 atm gave **38** in 82% yield. For another method, treatment of ketone **35** with Pd/C as the catalyst in reluxing acetic acid at 1 atm gave the key intermediate **38** in 59% yield. Then amino and phenol protection and Pictet–Spengler cyclomethylenation proceeded sequentially to give **40**, which deprotection afforded phenanthroquinuolizidine alkaloid **16** in 69% yield (**Figure 4**). Treatment of pyridine **37** with cyclopropylmethyl bromide obtained **41**, which was reduced using PtO_2_ as the catalyst in ethanol and conc. HCl (v/v 20∶1) at 50 atm to afford the key intermediate **42**. The Pictet–Spengler cyclomethylenation of **42** gave phenanthroquinuolizidine alkaloid **17** in 80% yield (**Figure 5**).

### Phytotoxic Activity

The phenanthroquinuolizidine alkaloids **1**–**24** were first tested for their phytotoxic activity against the test plant, and the results indicated that these natural product-based compounds showed no phytotoxic activity at 500 µg/mL.

### Antiviral Activity *In Vitro* And *In Vivo*

The commercial plant virucide Ningnanmycin, perhaps the most successful registered anti-plant viral agent, Ribavirin and (*R*)-antofine were used as the controls.

In order to investigate the subsituent effects of phenanthrene moiety, a series of racemic phenanthroquinolizidine alkaloids **1**, **4**, **7**, and **10**–**18** were designed, synthesized and evaluated for their antiviral activity (Table 1). To our delight, all the racemic phenanthroquinuolizidine alkaloids displayed higher antiviral activity than Ribavirin, of which cryptopleurine (**1**) and alkaloid **16** exhibited significantly higher activity than (*R*)-antofine and commercial Ningnanmycin, thus emerged as novel potent lead compounds for anti-TMV agents. The *in vitro* activity and *in vivo* activity of each alkaloid are about similar, which indicates that these alkaloids possess a good biological availability.

**Table 1:** *In vitro* and *in vivo* anti-TMV activity of racemic phenanthroquinolizidine alkaloids 1, 4, 7, and 10–18.

Compd.	Concn. (µg/mL)	*In vitro*	*In vivo*		
		Inhibition ratio (%)	Inactivation effect (%)	Curative effect (%)	Protection effect (%)
**1**	**500**	**75.4+2**	**73.1+1**	**71.5+2**	**73.8+1**
	**100**	**48.2+3**	**43.6+2**	**38.3+1**	**53.6+2**
**4**	500	41.4+5	45.0+3	45.9+4	42.5+8
	100	20.3+2	27.6+1	20.2+4	16.3+3
**7**	500	48.7+5	46.1+6	44.2+3	39.6+2
	100	20.3+3	25.2+7	27.1+4	20.2+5
**10**	500	46.9+2	41.8+8	42.7+6	38.7+3
	100	22.3+1	10.3+4	15.2+5	9.6+3
**11**	500	47.8+2	44.3+4	39.2+3	49.3+7
	100	22.4+4	9.8+3	12.6+2	18.2+6
**12**	500	46.4+2	43.2+1	40.8+1	48.2+4
	100	28.3+5	20.1+1	25.3+3	26.7+6
**13**	500	58.2+2	57.2+3	52.2+7	56.5+4
	100	30.1+1	22.1+5	20.1+6	26.2+4
**14**	500	50.5+3	52.1+5	46.6+7	49.2+1
	100	23.2+2	29.7+6	25.1+3	19.7+1
**15**	**500**	**70.3+2**	**68.2+1**	**66.6+1**	**64.3+2**
	**100**	**45.7+1**	**38.2+2**	**42.3+1**	**40.6+3**
**16**	**500**	**72.4+2**	**73.6+1**	**77.3+3**	**76.3+2**
	**100**	**57.6+2**	**51.4+1**	**55.2+1**	**55.6+2**
**17**	500	43.1+1	40.2+3	48.3+4	51.6+3
	100	25.2+3	20.1+3	28.1+2	28.4+4
**18**	500	66.6+7	55.4+3	61.4+5	60.2+6
	100	36.8+1	32.3+3	40.3+10	37.3+6
**(** ***R*** **)-Antofine**	**500**	**52.8+1**	**42.7+2**	**46.0+1**	**45.5+3**
	**100**	**27.4+2**	**15.3+2**	**19.4+1**	**20.4+4**
**Ribavirin**	**500**	**41.0+3**	**33.8+2**	**36.7+1**	**38.9+3**
	**100**	**19.6+5**	**9.8+2**	**8.2+1**	**7.5+2**
**Ningnanmycin**	**500**	**70.2+1**	**68.5+1**	**56.0+2**	**66.6+1**
	**100**	**23.9+2**	**37.7+3**	**18.9+1**	**25.2+2**

Cryptopleurine (**1**) exhibited significantly higher activity than alkaloid **7**, which indicated that the removal of methoxyl at 7-position of the phenanthrene moiety is beneficial for mataining excellent antiviral activity. Alkaloids **4** and **7** displayed about similar activity, which indicated that the removal of methoxyl at 2-position of the phenanthrene moiety is tolerant. The 6-position of the phenanthrene moiety, which is proposed to interact with TMV RNA [26], really is a sensitive site: removal of 6-O methyl increased the antiviral activity (antiviral activity: **16**>**1**; **18**>**2**), which indicated that the 6-position may be a hydrogen donor site; replacement of 6-O methyl with 6-O cyclopropyl methyl significantly decreased the antiviral activity (antiviral activity: **1**>**17**), which indicated that the steric effect is also a very important factor. Instead of 2,3-dimethoxyl or 6,7-dimethoxyl with methylenedioxyl is tolerant (antiviral activity: **7** ≈ **10** ≈ **11** ≈ **12**). However, replacement of 2,3-dimethoxyl or 6,7-dimethoxyl with ethylenedioxyl significantly increased the anti-TMV activity, especially for 6,7-dimethoxyl (antiviral activity: **15**>**13**>**14**>**7**).

Further to investigated the effect of C_14a_ chirality on anti-TMV activity, a series of chiral phenanthroquinolizidine alkaloids **2**, **3**, **5**, **6**, **8** and **9** were prepared and evaluated for their antiviral activity (Table 2). The (*R*)-phenanthroquinolizidine alkaloids exhibited significantly higher activity than their enantiomers (antiviral activity: **2**>**3**; **5**>**6**; **8**>**9**). The structure difference between (*R*)-cryptopleurine (**2**) and (*R*)-antofine lies in the “E” ring. (*R*)-Antofine with a 5-member “E” ring displayed inhibition activity of 42.7% inactivation effect, 46.0% curative effect and 45.5% protection effect at 500 µg/mL. However, (*R*)-cryptopleurine (**2**) with a 6-member “E” ring exhibited significantly higher *in vivo* antiviral activity than (*R*)-antofine even at 100 µg/mL. The results further indicated that although phenanthroquinolizidine and phenanthroindolizidine are structure analogs, their biological properties are different [22]. For (*R*)-phenanthroquinolizidine alkaloids: removal of 7-methoxyl increased antiviral activity (antiviral activity: **2**>**8**); removal of 2-methoxyl decreased antiviral activity (antiviral activity: **5**>**6**). However, the (*S*)-phenanthroquinolizidine alkaloids displayed about similar moderate antiviral activity (antiviral activity: **3** ≈ **6** ≈ **9**).

**Table 2:** *In vitro* and *in vivo* anti-TMV activity of chiral phenanthroquinolizidine alkaloids 2, 3, 5, 6, 8 and 9.

Compd.	Concn. (µg/mL)	*In vitro*	*In vivo*		
		Inhibitionratio (%)	Inactivationeffect (%)	Curativeeffect (%)	Protectioneffect (%)
**2**	**500**	**72.5+1**	**76.3+3**	**76.2+2**	**65.4+1**
	**100**	**55.6+2**	**52.4+1**	**50+1**	**41.8+2**
**3**	500	51.2+4	46.2+2	43.5+2	47.1+3
	100	25.3+1	17.8+5	13.4+3	22.2+6
**5**	500	51.8+4	45.6+1	49.4+1	47.2+2
	100	27.6+3	18.9+2	26.2+1	27.9+1
**6**	500	42.7+3	30.1+2	36.4+2	46.6+5
	100	20.0+1	16.2+3	25.9+3	18.0+2
**8**	500	64.6+3	61.5+1	60.0+1	57.8+5
	100	33.3+1	27.2+2	25.3+1	35.6+3
**9**	500	45.2+5	40.1+3	41.8+1	36.9+2
	100	10.3+3	21.3+12	22.4+7	18.5+9
**(** ***R*** **)-Antofine**	**500**	**52.8+1**	**42.7+2**	**46.0+1**	**45.5+3**
	**100**	**27.4+2**	**15.3+2**	**19.4+1**	**20.4+4**
**Ribavirin**	**500**	**41.0+3**	**33.8+2**	**36.7+1**	**38.9+3**
	**100**	**19.6+5**	**9.8+2**	**8.2+1**	**7.5+2**
**Ningnanmycin**	**500**	**70.2+1**	**68.5+1**	**56.0+2**	**66.6+1**
	**100**	**23.9+2**	**37.7+3**	**18.9+1**	**25.2+2**

In order to investigated the effect of 15-hydroxyl on antiviral activity, a series of 15-hydroxyphenanthroquinolizidine alkaloids **19**–**24** were prepared and evaluated for their antiviral activity (Table 3). The bioassay results showed that the 15-hydroxyphenanthroquinolizidine alkaloids also displayed good to excellent *in vivo* anti-TMV activity, of which alkaloids **19**, **20**, **22** and **24** exhibited signigicantly higher antiviral activity than Ribavirin, and alkaloids **19** and **22** displayed higher *in vivo* activity than Ningnanmycin at 100 µg/mL. The introduction of hydroxy group at 15-position significantly increased antiviral activity for (*S*)-phenanthroquinolizidine alkaloids (antiviral activity: **2**>**19**; **5**>**21**; **8**>**23**), but decreased antiviral activity for (*R*)-phenanthroquinolizidine alkaloids (antiviral activity: **20**>**3**; **22**>**6**; **24**>**9**). The (*S*,*S*)-enantiomers **22** and **24** exhibited significantly higher antiviral activity than their corresponding enantiomers **21** and **23**. However, the (*S*,*S*)-enantiomer **20** showed a lower activity than its enantiomer **19**. For (14a*S*,15*S*)-phenanthroquinolizidine alkaloids: removal of 7-methoxyl decreased antiviral activity (antiviral activity: **20**<**24**); removal of 2-methoxyl increased antiviral activity (antiviral activity: **22**>**24**). For (14a*R*,15*R*)-phenanthroquinolizidine alkaloids, removal of 7-methoxyl or 2-methoxyl all increased antiviral activity (antiviral activity: **19**>**21**>**23**).

**Table 3:** *In vitro* and *in vivo* anti-TMV activity of 15-hydroxyphenanthroquinolizidine alkaloids 19–24.

Compd.	Concn. (µg/mL)	*In vitro*	*In vivo*		
		Inhibitionratio (%)	Inactivationeffect (%)	Curativeeffect (%)	Protectioneffect (%)
**19**	**500**	**52.0+1**	**53.7+2**	**57.3+4**	**58.9+2**
	**100**	**28.8+1**	**40.4+3**	**35.8+2**	**31.0+1**
**20**	500	51.7+3	51.2+2	53.4+2	50.0+1
	100	28.7+4	18.7+3	17.5+1	23.4+2
**21**	500	46.4+5	44.9+3	46.7+4	45.2+1
	100	18.5+2	17.0+2	20.2+4	21.7+5
**22**	**500**	**65.3+3**	**60.3+1**	**56.7+2**	**63.8+3**
	**100**	**40.0+2**	**37.9+1**	**32.3+2**	**38.5+3**
**23**	500	35.9+1	39.6+2	40.3+3	42.7+2
	100	16.6+3	5.8+2	13.1+4	10.2+2
**24**	500	52.5+5	57.6+3	55.7+2	52.4+4
	100	34.0+3	26.0+1	26.8+2	32.6+4
**(** ***R*** **)-Antofine**	**500**	**52.8+1**	**42.7+2**	**46.0+1**	**45.5+3**
	**100**	**27.4+2**	**15.3+2**	**19.4+1**	**20.4+4**
**Ribavirin**	**500**	**41.0+3**	**33.8+2**	**36.7+1**	**38.9+3**
	**100**	**19.6+5**	**9.8+2**	**8.2+1**	**7.5+2**
**Ningnanmycin**	**500**	**70.2+1**	**68.5+1**	**56.0+2**	**66.6+1**
	**100**	**23.9+2**	**37.7+3**	**18.9+1**	**25.2+2**

## Materials and Methods

### Synthesis

Reagents were purchased from commercial sources and were used as received. All anhydrous solvent were dried and purified by standard techniques just before use. Reaction progress was monitored by thin-layer chromatography on silica gel GF_254_ with detection by UV. Please see supporting information for the melting points, ^1^H NMR spectra, ^13^C NMR spectra, elemental analyses or high-resolution mass spectra of the synthesized compounds **10**–**17** and **26**–**42**.

### General Procedure for the Synthesis of Methanones 26a–f

To a stirred mixture of 2-bromopyridine (80 mmol) in THF (300 mL) was added *n-*BuLi (32 mL, 80 mmol, 2.5 M in hexane solution) at −78°C over 30 min. The brown solution was stirred at –78°C for 4 h and the acid **25** (20 mmol) was added at once. The mixture was stirred at –78°C for 2 h and then warmed to room temperature for 6 h, then quenched with saturated aqueous NH_4_Cl (300 mL) and extracted with CH_2_Cl_2_ (3×200 mL). The organic layer was dried over anhydrous Na_2_SO_4_, filtered and concentrated *in vacuo*. The residue was added dissolved in methanol (50 mL), and the precipitate was filtered and washed with methanol (50 mL) to give methanone **26**.

### General Procedure for the Synthesis of Alcohol Amines 27a–e

A mixture of **26** (5 mmol), acetic acid (150 mL), trifluoroacetic acid (30 mL) and PtO_2_·H_2_O (50 mg) was stirred for 36 h at a pressure of 50 atm under hydrogen. The mixture was filtered, and the filtrate was evaporated to dryness *in vacuo*. The residue was treated with aqueous NaOH and extracted with CH_2_Cl_2_ (3×100 mL). The combined organic extracts were dried over anhydrous Na_2_SO_4_, filtered and concentrated *in vacuo*. The residue was purified by flash column chromatography on silica gel with CH_2_Cl_2_/CH_3_OH (12∶1) as eluant to afford **27**.

### General Procedure for the Synthesis of Piperidines 28a–e

A mixture of **27** (5 mmol), trifluoroacetic acid (120 mL) and triethylsilane (6 mmol) was refluxed for 8 h. The reaction mixture was concentrated to dryness under reduced pressure. The residue was treated with aqueous NaOH and extracted with CH_2_Cl_2_ (3×100 mL). The combined organic layer was dried over anhydrous Na_2_SO_4_, filtered and concentrated *in vacuo*. The residue was purified by flash column chromatography on silica gel with CH_2_Cl_2_/CH_3_OH (10∶1) as eluant to afford **28**.

### General Procedure for the Synthesis of Phenanthroquinolizidine Alkaloids 10–14

To a solution of piperidine **28** (3 mmol) in ethanol (90 mL) was added a 37% formaldehyde solution (20 mL) over 10 min, and then conc. HCl (2 mL) was added. The reaction mixture was refluxed for 24 h in the dark, then evaporated under reduced pressure to remove most of the ethanol. The residue was filtered and washed with ethanol (5 mL) to give a white solid. The solid was dissolved in CH_2_Cl_2_ and washed with aqueous NaOH. The organic layer was dried over anhydrous MgSO_4_, filtered and concentrated *in vacuo* to afford alkaloids **10**–**14**.

### Synthesis of 2-[(2,3-Ethylenedioxy-6,7-dimethoxyphenanthren-9-yl)methyl]pyridine (29)

A mixture of **26f** (0.5 mmol), acetic acid (15 mL), trifluoroacetic acid (3.8 mL) and PtO_2_·H_2_O (3 mg) was stirred for 24 h at a pressure of 50 atm under hydrogen. The mixture was filtered, and the filtrate was evaporated to dryness *in vacuo*. The residue was treated with aqueous NaOH and extracted with CH_2_Cl_2_ (3×20 mL). The combined organic extracts were dried over anhydrous Na_2_SO_4_, filtered and concentrated *in vacuo*. The residue was purified by flash column chromatography on silica gel with CH_2_Cl_2_/EtOAc (6∶1) as eluant to give **29** (0.1 g, 53%) as a white powder.

### Synthesis of 2-[(2,3-Ethylenedioxy-6,7-dimethoxyphenanthren-9-yl)methyl]piperidine (30)

A mixture of **29** (5 mmol), acetic acid (150 mL), trifluoroacetic acid (30 mL) and PtO_2_·H_2_O (10 mg) was stirred for 24 h at a pressure of 50 atm under hydrogen. The mixture was filtered, and the filtrate was evaporated to dryness *in vacuo*. The residue was treated with aqueous NaOH and extracted with CH_2_Cl_2_ (3×100 mL). The combined organic extracts were dried over anhydrous Na_2_SO_4_, filtered and concentrated *in vacuo*. The residue was purified by flash column chromatography on silica gel with CH_2_Cl_2_/CH_3_OH (2∶1) as eluant to give **30** (1.8 g, 92%) as a white powder.

### Synthesis of 6,7-Ethylenedioxy-2,3-dimethoxyphenanthro[9,10-b]quinolizidine (15)

Compound **15** was synthesized from **30** (3 mmol) by using a similar procedure for the synthesis of alkaloids **10**–**14**, white powder.

### Synthesis of (*E*)-Methyl 2-(3,4-dimethoxyphenyl)-3-(4-hydroxyphenyl)acrylate (31)

A mixture of 2-(3,4-dimethoxyphenyl)acetic acid (0.1 mol), 4-hydroxybenzaldehyde (0.1 mol), acetic anhydride (40 mL), and triethylamine (20 mL) was refluxed for 20 h with the exclusion of moisture. The solution was cooled to room temperature, then water (60 mL) was added, and the mixture was stirred for 1 h. The mixture was then poured into aqueous potassium carbonate (70.0 g in 160 mL water) and refluxed until nearly all the gummy material was dissolved. The solution obtained was cooled, extracted with ether (3×30 mL), and carefully acidified with concentrated hydrochloric acid (pH 4–5) to produce a white precipitate. The solid was collected and dried, then dissolved in methanol (600 mL). To the vigorously stirred methanol solution was added concentrated H_2_SO_4_ (4 g) dropwisely. The mixture was refluxed for 20 h with the exclusion of moisture, then concentrated *in vacuo*. The residue was purified by flash column chromatography on silica gel with EtOAc/petroleum ether (1∶2) as eluant to afford ester **31** (16.1 g, 51%) as a white powder.

### Synthesis of Methyl 3-hydroxy-6,7-dimethoxyphenanthrene-9-carboxylate (32)

To a stirred mixture of FeCl_3_ (35.8 mmol) in CH_2_Cl_2_ (300 mL) was added dropwisely a solution of ester **31** (0.03 mol) in CH_2_Cl_2_ (500 mL) over 2 h under nitrogen at 0–5°C. The mixture was warmed to room temperature for 4 h, and quenched with methanol (30 mL). The residue was washed with H_2_O (4×200 mL) and brine (2×200 mL), dried over anhydrous Na_2_SO_4_, filtered, and concentrated *in vacuo*, then purified by flash column chromatography on silica gel with EtOAc/petroleum ether (1∶3) as eluant to obtain **32** (4.8 g, 51%) as a yellow powder.

### Synthesis of Methyl 3-(benzyloxy)-6,7-dimethoxyphenanthrene-9-carboxylate (33)

A stirred mixture of ester **32** (0.02 mol), benzyl bromide (0.03 mol) and K_2_CO_3_ (0.04 mol) in acetone (350 mL) was refluxed for 6 h, and quenched with H_2_O (200 mL). The mixture was cooled to room temperature and extracted with CH_2_Cl_2_ (3×200 mL). The combined organic layer was washed with brine (200 mL) and dried over anhydrous Na_2_SO_4_, filtered and concentrated *in vacuo*. The residue was purified by flash column chromatography on silica gel with EtOAc/petroleum ether (1∶2) as eluant to give **33** (7.4 g, 92%) as a white powder.

### Synthesis of 3-(Benzyloxy)-6,7-dimethoxyphenanthrene-9-carboxylic acid (34)

A solution of ester **33** (0.01 mol) and KOH (0.05 mol) in H_2_O (140 mL) and CH_3_OH (140 mL) was refluxed for 8 h, and cooled to room temperature. The solution obtained was cooled, extracted with ether (3×100 mL), and carefully acidified with concentrated hydrochloric acid (pH 4–5) to produce a white precipitate, filtrated and dried to give acid **34** (3.8 g, 98%) as a white powder.

### Synthesis of (3-(Benzyloxy)-6,7-dimethoxyphenanthren-9-yl)(pyridin-2-yl)methanone (35)

Compound **35** was synthesized from **34** (20 mmol) by using a similar procedure for the synthesis of methanone **26**, yellow powder (5.4 g, 60%).

### Synthesis of (3-Hydroxy-6,7-dimethoxyphenanthren-9-yl)(pyridin-2-yl)methanol (36)

A mixture of methanone **35** (4 mmol) and 10% Pd/C (0.1 g) in CH_3_OH (600 mL) was refluxed for 24 h under hydrogen. The solution was cooled to room temperature, filtrated and concentrated *in vacuo*. The residue was recrystallized from CH_2_Cl_2_ and petroleum ether to give alcohol **36** (1.2 g, 83%) as a white powder.

### Synthesis of 2-[(3-Hydroxy-6,7-dimethoxyphenanthren-9-yl)methyl]pyridine (37)

A mixture of **36** (1 mmol), trifluoroacetic acid (60 mL) and triethylsilane (10 mL) was refluxed for 8 h. The reaction mixture was concentrated to dryness under reduced pressure. The residue was treated with aqueous NaOH and extracted with CH_2_Cl_2_ (3×50 mL). The combined organic layer was dried over anhydrous Na_2_SO_4_, filtered and concentrated *in vacuo*. The residue was purified by flash column chromatography on silica gel with CH_2_Cl_2_/EtOAc (2∶1) as eluant to afford **37** (0.27 g, 79%) as a white powder.

### Synthesis of 2-[(3-Hydroxy-6,7-dimethoxyphenanthren-9-yl)methyl]piperidine (38)

#### Method A

Compound **38** was synthesized from **37** (3 mmol) as a white powder (0.87 g, 82%) by using a similar procedure for the synthesis of compound **30** except for under hydrogen at a pressure of 10 atm.

#### Method B

A mixture of methanone **35** (4 mmol) and 10% Pd/C (0.16 g) in acetic acid (40 mL) was stirred at room temperature for 24 h, and then refluxed for 24 h under hydrogen. The mixture was cooled to room temperature, filtrated and concentrated *in vacuo*. The residue was treated with aqueous NaOH and extracted with CH_2_Cl_2_ (3×100 mL). The combined organic layer was dried over anhydrous Na_2_SO_4_, filtered and concentrated *in vacuo*. The residue was purified by flash column chromatography on silica gel with CH_2_Cl_2_/CH_3_OH (12∶1) as eluant to afford **38** (0.83 g, 59%) as a white powder.

### Synthesis of 2-[(3-(Benzyloxy)-6,7-dimethoxyphenanthren-9-yl)methyl]piperidine (39)

A mixture of **38** (1 mmol), (Boc)_2_O (9.3 mmol) and saturated NaHCO_3_ solution (1.5 mL) in THF (40 mL) was stirred at room temperature for 3 h, then extracted with CH_2_Cl_2_ (3×50 mL). The combined organic layer was washed with brine (2×50 mL), dried over anhydrous Na_2_SO_4_, filtrated and concentrated *in vacuo*. The residue was dissolved in acetone. To the solution was added benzyl bromide (4.2 mmol) and K_2_CO_3_ (5.8 mmol). The mixture was refluxed for 3 h under nitrogen, and quenched with H_2_O (50 mL), extracted with CH_2_Cl_2_ (3×50 mL). The combined organic layer was washed with brine (2×50 mL), dried over anhydrous Na_2_SO_4_, filtered and concentrated *in vacuo*. The residue was recrystallized from ether acetate and petroleum ether to give a white powder. A mixture of the powder in 1,4-dioxane (20 mL) was stirred for 45 min at 0°C under HCl gas atmosphere. The reaction mixture was concentrated to dryness under reduced pressure. The residue was treated with aqueous NaOH and extracted with CH_2_Cl_2_ (3×50 mL). The combined organic layer was dried over anhydrous Na_2_SO_4_, filtered and concentrated *in vacuo*. The residue was recrystallized from ethanol to afford compound **39** (0.16 g, 36%) as a white powder.

### Synthesis of 6-Benzyloxy-2,3-dimethoxyphenanthro[9,10-b]quinolizidine (40)

Compound **40** was synthesized from **39** (3 mmol) by using a similar procedure for the synthesis of alkaloids **10**–**14**, white powder (1.1 g, 80%).

### Synthesis of 6-Hydroxy-2,3-dimethoxyphenanthro[9,10-b]quinolizidine (16)

A mixture of compound **40** (0.08 mmol) and 10% Pd/C (0.1 g) in THF (50 mL) was stirred for 36 h under hydrogen in the dark. The mixture was filtered and concentrated *in vacuo* to afford alkaloid **16** (0.02 g, 69%) as a light pink powder.

### Synthesis of 2-[(3-Cyclopropylmethoxy-6,7-dimethoxyphenanthren-9-yl)methyl]pyridine (41)

A mixture of pyridine **37** (2.8 mmol) and Cs_2_CO_3_ (3.7 mmol) in DMF (20 mL) was stirred for 1 h at room temperature. To the result solution was added cyclopropylmethyl bromide (3.3 mmol). The mixture was stirred for 10 h at room temperature, and quenched with H_2_O (100 mL), then extracted with CH_2_Cl_2_ (3×50 mL). The combined organic layer was washed with H_2_O (6×50 mL) and brine (3×50 mL), dried over anhydrous MgSO_4_, filtered and concentrated *in vacuo*. The residue was purified by flash column chromatography on silica gel with CH_2_Cl_2_/EtOAc (6∶1) as eluant to afford **41** (1.0 g, 83%) as a white powder.

### Synthesis of 2-[(3-Cyclopropylmethoxy-6,7-dimethoxyphenanthren-9-yl)methyl]piperidine (42)

A mixture of **41** (1 mmol), conc. HCl solution (5 mL), ethanol (100 mL) and PtO_2_·H_2_O (0.1 g) was stirred for 36 h at a pressure of 50 atm under hydrogen. The mixture was filtered, and the filtrate was evaporated to dryness *in vacuo*. The residue was treated with aqueous NaOH and extracted with CH_2_Cl_2_ (3×100 mL). The combined organic extracts were dried over anhydrous Na_2_SO_4_, filtered and concentrated *in vacuo*. The residue was purified by flash column chromatography on silica gel with CH_2_Cl_2_/CH_3_OH (12∶1) as eluant to give **42** (0.3 g, 83%) as a white powder.

### Synthesis of 6-Cyclopropylmethoxy-2,3-dimethoxyphenanthro[9,10-b]quinolizidine (17)

Compound **17** was synthesized from **42** (3 mmol) by using a similar procedure for the synthesis of alkaloids **10**–**14**, white powder (0.87 g, 80%).

### Antiviral Biological Assay

The anti-TMV activity of the synthesized pure compounds was tested using our previously reported method [30].

#### Antiviral activity of compounds against TMV *in vitro*

Fresh leaf of the 5–6 growth stage of tobacco (*Nicotiana tabacum var Xanthi nc*) inoculated by the juice-leaf rubbing method (concentration of TMV is 5.88 × 10^-2^ *µ*g/mL) was cut into halves along the main vein. The halves were immersed into the solution of 500 µg/mL of the compounds and double distilled water for 20 min, respectively, and then cultured at 25°C for 72 h. Each compound was replicated at least three times.

#### Protective effect of compounds against TMV *in vivo*

The compound solution was smeared on the left side and the solvent serving as control on the right side of growing tobacco (*Nicotiana tabacum var Xanthi nc*) leaves of the same ages. The leaves were then inoculated with the virus after 12 h. A brush was dipped in TMV of 6 × 10^−3^ mg/mL to inoculate the leaves, which were previously scattered with silicon carbide. The leaves were then washed with water and rubbed softly along the nervature once or twice. The local lesion numbers appearing 3–4 days after inoculation were counted. There are three replicates for each compound.

#### Inactivation effect of compounds against TMV *in vivo*

The virus was inhibited by mixing with the compound solution at the same volume for 30 min. The mixture was then inoculated on the left side of the leaves of tobacco (*Nicotiana tabacum var Xanthi nc*), whereas the right side of the leaves was inoculated with the mixture of solvent and the virus for control. The local lesion numbers were recorded 3–4 days after inoculation. There are three replicates for each compound.

#### Curative effect of compounds against TMV *in vivo*

Growing leaves of tobacco (*Nicotiana tabacum var Xanthi nc*) of the same ages were selected. TMV (concentration of 6.0 × 10^−3^ mg/mL) was dipped and inoculated on the whole leaves. Then the leaves were washed with water and dried. The compound solution was smeared on the left side, and the solvent was smeared on the right side for control. The local lesion numbers were then counted and recorded 3–4 days after inoculation. There are three replicates for each compound.

The *in vitro* and *in vivo* inhibition rates of the compound were then calculated according to the following formula (“av” means average, and controls were not treated with compound).

Inhibition rate (%) = [(av local lesion no. of control − av local lesion no. of drug-treated)/av local lesion no. of control] × 100%.

### Conclusion

In summary, based on the literature report and our previous work, a series of phenanthroquinolizidine alkaloids **1**–**24** were designed, prepared and evaluated for their anti-TMV activity for the first time. The *in vitro* and *in vivo* antiviral bioassays showed that most of these alkaloids exhibited good to excellent *in vivo* anti-TMV activity, of which compounds **1**, **2**, **15** and **16** displayed significantly higher activity than (*R*)-antofine and commercial Ningnanmycin at the same test condition. The *in vitro* activity and *in* *vivo* activity of each alkaloid are about similar, which indicates that these alkaloids possess a good biological availability. The introduction of 6-hydroxyl, which is proposed to interact with TMV RNA, did increased anti-TMV activity. The 14a*R*-configuration was confirmed to be the preferred antiviral configuration for phenanthroquinolizidine alkaloids. Introduction of hydroxy group at 15-position of phenanthroquinolizidine alkaloids increased antiviral activity for *S*-configuration but decreased activity for *R*-configuration. Present study provides fundamental support for development and optimization of phenanthroquinolizidine alkaloids as potential inhibitors of plant virus. To the best of our knowledge, this is the first report on anti-TMV activity of phenanthroquinolizidine alkaloids. Further studies on mode of action are currently underway in our laboratories.

## Supporting Information

Figure S1 **^1^H NMR and ^13^C NMR spectra of the synthesized compounds 10–17 and 26–42.** ^1^H NMR spectra: **26a**–**f**, **27a**–**e**, **28a**–**e**, **10**–**17** and **29**–**42**. ^13^C NMR spectra: **26a**–**f**, **27c**–**e**, **28a**, **28c**, **28d**, **10**–**17** and **29**–**42**.
(DOC)

Text S1 **Experimental data of the synthesized compounds 10–17 and 26–42.**
(DOC)
