# Supplementary material for: First Discovery and Stucture-Activity Relationship Study of Phenanthroquinolizidines as Novel Antiviral Agents against Tobacco Mosaic Virus (TMV)
Source: PLoS One. 2012 Dec 28;7(12):e52933. doi: 10.1371/journal.pone.0052933 (PMC3532156; doi:10.1371/journal.pone.0052933)
Supplement: Figure S1 — 1H NMR and 13C NMR spectra of the synthesized compounds 10–17 and 26–42. 1H NMR spectra: 26a–f, 27a–e, 28a–e, 10–17 and 29–42. 13C NMR spectra: 26a–f, 27c–e, 28a, 28c, 28d, 10–17 and 29–42. (DOC) [file pone.0052933.s001.doc]

***Electronic supplementary information (ESI)***

First Discovery and Structure-Activity Relationship Study of Phenanthroquinolizidines as Novel Antiviral Agents Against *Tobacco Mosaic Virus* (TMV)

Ziwen Wang, Anzheng Feng, Mingbo Cui, Yuxiu Liu, Lizhong Wang, Qingmin Wang*

State Key Laboratory of Elemento-Organic Chemistry, Research Institute of Elemento-Organic Chemistry, Nankai University, Tianjin 300071, China.

Tel: +86-22-23503952; fax: +86-22-23503952; e-mail: [wangqm@nankai.edu.cn](mailto:wangqm@nankai.edu.cn)

**Contents**

^1^H NMR, ^13^C NMR spectra of **26a…………………………..………………………………………………….…………** S3

^1^H NMR, ^13^C NMR spectra of **26b……………………………..……………………………………………….…………** S4

^1^H NMR, ^13^C NMR spectra of **26c……………………………..………………………………………………….…………** S5

^1^H NMR, ^13^C NMR spectra of **26d…………………………..………………………………………………….…………** S6

^1^H NMR, ^13^C NMR spectra of **26e…………………………..………………………………………………….…………** S7

^1^H NMR, ^13^C NMR spectra of **26f…………………………..………………………………………………….…………** S8

^1^H NMR spectra of **27a** and **27b…………….…………………………………………………………………………………**S9

^1^H NMR, ^13^C NMR spectra of **27c…………………………..………………………………………………….…………** S10

^1^H NMR, ^13^C NMR spectra of **27d……………………………..……………………………………………….…………** S11

^1^H NMR, ^13^C NMR spectra of **27e……………………………..………………………………………………….…………** S12

^1^H NMR, ^13^C NMR spectra of **28a…………………………..………………………………………………….…………** S13

^1^H NMR spectra of **28b** and **28c…………….…………………………………………………………………………………**S14

^13^C NMR spectrum of **28c** and ^1^H NMR spectrum of **28d……………………………………………………**S15

^13^C NMR spectrum of **28d** and ^1^H NMR spectrum of **28e……………………………………………………**S16

^1^H NMR, ^13^C NMR spectra of **10…………………………..………………………………………………….…………** S17

^1^H NMR, ^13^C NMR spectra of **11…………………………..………………………………………………….…………** S18

^1^H NMR, ^13^C NMR spectra of **12…………………………..………………………………………………….…………** S19

^1^H NMR, ^13^C NMR spectra of **13……………………………..……………………………………………….…………** S20

^1^H NMR, ^13^C NMR spectra of **14……………………………..………………………………………………….…………** S21

^1^H NMR, ^13^C NMR spectra of **29…………………………..………………………………………………….…………** S22

^1^H NMR, ^13^C NMR spectra of **30…………………………..………………………………………………….…………** S23

^1^H NMR, ^13^C NMR spectra of **15…………………………..………………………………………………….…………** S24

^1^H NMR, ^13^C NMR spectra of **31…………………………..………………………………………………….…………** S25

^1^H NMR, ^13^C NMR spectra of **32……………………………..……………………………………………….…………** S26

^1^H NMR, ^13^C NMR spectra of **33……………………………..………………………………………………….…………** S27

^1^H NMR, ^13^C NMR spectra of **34…………………………..………………………………………………….…………** S28

^1^H NMR, ^13^C NMR spectra of **35…………………………..………………………………………………….…………** S29

^1^H NMR, ^13^C NMR spectra of **36…………………………..………………………………………………….…………** S30

^1^H NMR, ^13^C NMR spectra of **37…………………………..………………………………………………….…………** S31

^1^H NMR, ^13^C NMR spectra of **38……………………………..……………………………………………….…………** S32

^1^H NMR, ^13^C NMR spectra of **39……………………………..………………………………………………….…………** S33

^1^H NMR, ^13^C NMR spectra of **40…………………………..………………………………………………….…………** S34

^1^H NMR, ^13^C NMR spectra of **16…………………………..………………………………………………….…………** S35

^1^H NMR, ^13^C NMR spectra of **41…………………………..………………………………………………….…………** S36

^1^H NMR, ^13^C NMR spectra of **42…………………………..………………………………………………….…………** S37

^1^H NMR, ^13^C NMR spectra of **17……………………………..……………………………………………….…………** S38

**26a**

**26b**

**26c**

**26d**

**26e**

**26f**

**27a and 27b**

**27c**

**27d**

**27e**

**28a**

**28b and 28c**

**28c and 28d**

**28d and 28e**

**10**

**11**

**12**

**13**

**14**

**29**

**30**

**15**

**31**

**32**

**33**

**34**

**35**

**36**

**37**

**38**

**39**

**40**

**16**

**41**

**42**

**17**
