# Supplementary material for: First Discovery and Stucture-Activity Relationship Study of Phenanthroquinolizidines as Novel Antiviral Agents against Tobacco Mosaic Virus (TMV)
Source: PLoS One. 2012 Dec 28;7(12):e52933. doi: 10.1371/journal.pone.0052933 (PMC3532156; doi:10.1371/journal.pone.0052933)
Supplement: Text S1 — Experimental data of the synthesized compounds 10–17 and 26–42. (DOC) [file pone.0052933.s002.doc]

***Electronic supplementary information (ESI)***

First Discovery and Structure-Activity Relationship Study of Phenanthroquinolizidines as Novel Antiviral Agents Against *Tobacco Mosaic Virus* (TMV)

Ziwen Wang, Anzheng Feng, Mingbo Cui, Yuxiu Liu, Lizhong Wang, Qingmin Wang*

State Key Laboratory of Elemento-Organic Chemistry, Research Institute of Elemento-Organic Chemistry, Nankai University, Tianjin 300071, China.

Tel: +86-22-23503952; fax: +86-22-23503952; e-mail: [wangqm@nankai.edu.cn](mailto:wangqm@nankai.edu.cn)

**Contents**

**Experimental data………………………………………………………………..………………………………………………….…………** S2

**Experimental data**

**General**. Melting points were determined using an X-4 binocular microscope melting point apparatus (Beijing Tech Instruments Co., Beijing, China) and the thermometer was uncorrected. ^1^H NMR spectra were obtained by using Bruker AV 400, Bruker AV300 and a Varian Mercury Plus 400 MHz spectrometer. Chemical shifts (*δ*) were given in parts per million (ppm) and were measured downfield from internal tetramethylsilane. ^13^C NMR spectra were recorded by using Bruker AV 400 (100 MHz) and Bruker AV300 (75 MHz) with CDCl_3_ or DMSO-*d*_6_ as a solvent. Chemical shifts (*δ*) were reported in parts per million using the solvent peak. Elemental analyses were determined on a Yanaco C, H, N Corder MT-3 elemental analyzer. High-resolution mass spectra were obtained with an FT-ICR MS spectrometer (Ionspec, 7.0 T).

*(2,3-Methylenedioxy-6,7-methylenedioxyphenanthren-9-yl)(pyridin-2-yl)methanone* *(****26a****)*. Yield 64%; yellow powder; mp 280–282 °C; ^1^H NMR (400 MHz, CDCl_3_) *δ* 6.10 (s, 2H), 6.12 (s, 2H), 7.14 (s, 1H), 7.54–7.57 (m, 1H), 7.77 (s, 1H), 7.79 (s, 2H), 7.83 (s, 1H), 7.97–8.00 (m, 1H), 8.15–8.17 (m, 1H), 8.75 (d, *J* = 4.4 Hz, 1H); ^13^C NMR (100 MHz, CDCl_3_) *δ* 100.54, 101.45, 101.56, 104.11, 106.44, 124.80, 125.36, 125.68, 126.44, 127.27, 128.84, 130.65, 131.07, 147.34, 147.88, 148.08, 149.11, 149.88, 155.82, 196.30; HRMS (ESI) calcd for C_22_H_14_NO_5_ (M+H)^+^ 372.0866, found 372.0871.

*(2,3-Dimethoxy-6,7-methylenedioxyphenanthren-9-yl)(pyridin-2-yl)methanone* *(****26b****)*. Yield 55%; yellow powder; mp 214–215 °C; ^1^H NMR (400 MHz, CDCl_3_) *δ* 4.01 (s, 3H), 4.15 (s, 3H), 6.13 (s, 2H), 7.19 (s, 1H), 7.54–7.57 (m, 1H), 7.84 (s, 1H), 7.85 (s, 1H), 7.89 (s, 1H), 7.97–8.01 (m, 2H), 8.17–8.19 (m, 1H), 8.78 (d, *J* = 4.0 Hz, 1H); ^13^C NMR (100 MHz, CDCl_3_) *δ* 55.93, 56.00, 100.30, 101.45, 102.66, 104.33, 109.08, 124.33, 124.81, 125.21, 126.31, 126.80, 127.16, 130.70, 130.79, 137.02, 147.73, 147.99, 149.07, 149.13, 151.08, 156.08, 196.30; HRMS (ESI) calcd for C_23_H_18_NO_5_ (M+H)^+^ 388.1179, found 388.1186.

*(2,3-Methylenedioxy-6,7-dimethoxyphenanthren-9-yl)(pyridin-2-yl)methanone* *(****26c****)*. Yield 52%; yellow powder; mp 243–244 °C; ^1^H NMR (400 MHz, CDCl_3_) *δ* 3.97 (s, 3H), 4.14 (s, 3H), 6.14 (s, 2H), 7.18 (s, 1H), 7.56–7.59 (m, 1H), 7.80 (s, 1H), 7.84 (s, 1H), 7.88 (s, 1H), 7.98–8.02 (m, 2H), 8.14–8.16 (m, 1H), 8.78 (d, *J* = 4.4 Hz, 1H); ^13^C NMR (100 MHz, CDCl_3_) *δ* 55.77, 55.86, 100.34, 101.58, 102.81, 106.53, 106.72, 124.20, 124.86, 125.46, 125.75, 126.28, 128.62, 129.91, 131.64, 137.12, 147.17, 149.06, 149.29, 149.36, 150.00, 156.21, 196.31; HRMS (ESI) calcd for C_23_H_18_NO_5_ (M+H)^+^ 388.1179, found 388.1185.

*(2,3-Ethylenedioxy-6,7-ethylenedioxyphenanthren-9-yl)(pyridin-2-yl)methanone* *(****26d****)*. Yield 59%; yellow powder; mp 238–240 °C; ^1^H NMR (400 MHz, CDCl_3_) *δ* 4.34–4.40 (m, 8H), 7.24 (s, 1H), 7.50–7.53 (m, 1H), 7.70 (s, 1H), 7.867–7.873 (m, 2H), 7.91–7.96 (m, 2H), 8.10–8.12 (m, 1H), 8.74 (d, *J* = 4.4 Hz, 1H); ^13^C NMR (100 MHz, CDCl_3_) *δ* 64.36, 64.41, 64.60, 64.64, 109.40, 109.46, 113.29, 115.78, 124.07, 124.69, 124.72, 125.89, 126.26, 127.11, 130.79, 131.04, 136.92, 143.51, 14374, 143.86, 145.65, 149.15, 156.00, 196.14; Anal. Calcd for C_24_H_17_NO_5_: C, 72.17; H, 4.29; N, 3.51. Found: C, 72.29; H, 4.45; N, 3.75.

*(6,7-Ethylenedioxy-2,3-dimethoxyphenanthren-9-yl)(pyridin-2-yl)methanone* *(****26e****)*. Yield 60%; yellow powder; mp 209–210 °C; ^1^H NMR (400 MHz, CDCl_3_) *δ* 3.98 (s, 3H), 4.11 (s, 3H), 4.36–4.41 (m, 4H), 7.15 (s, 1H), 7.49–7.52 (m, 1H), 7.79 (s, 1H), 7.80 (s, 1H), 7.91–7.98 (m, 3H), 8.12–8.14 (m, 1H), 8.74 (d, *J* = 4.8 Hz, 1H); ^13^C NMR (100 MHz, CDCl_3_) *δ* 55.87, 55.95, 64.39, 64.62, 102.86, 108.88, 109.42, 113.17, 124.01, 124.29, 124.70, 125.92, 126.16, 126.60, 130.21, 131.31, 136.90, 143.75, 143.86, 143.92, 149.06, 150.98, 156.17, 196.02; Anal. Calcd for C_24_H_19_NO_5_: C, 71.81; H, 4.77; N, 3.49. Found: C, 71.99; H, 4.93; N, 3.74.

*(2,3-Ethylenedioxy-6,7-dimethoxyphenanthren-9-yl)(pyridin-2-yl)methanone* *(****26f****)*. Yield 66%; yellow powder; mp 216–218 °C; ^1^H NMR (400 MHz, CDCl_3_) *δ* 3.94 (s, 3H), 4.10 (s, 3H), 4.37–4.43 (m, 4H), 7.29 (s, 1H), 7.49–7.52 (m, 1H), 7.79 (s, 1H), 7.82 (s, 1H), 7.91–7.97 (m, 3H), 8.10–8.12 (m, 1H), 8.74 (d, *J* = 5.2 Hz, 1H); ^13^C NMR (400 MHz, CDCl_3_) *δ* 54.71, 54.79, 63.29, 63.64, 75.77, 76.09, 76.41, 102.00, 105.83, 107.93, 114.73, 122.69, 123.69, 123.77, 124.14, 125.16, 126.68, 129.10, 130.55, 135.90, 142.39, 144.80, 148.08, 155.28, 195.36; Anal. Calcd for C_24_H_19_NO_5_: C, 71.81; H, 4.77; N, 3.49. Found: C, 71.96; H, 4.98; N, 3.64.

*(2,3-Methylenedioxy-6,7-methylenedioxyphenanthren-9-yl)(piperidin-2-yl)methanol (****27a****)*. Yield 51%; white powder; mp 226–228 °C; ^1^H NMR (400 MHz, CDCl_3_) *δ* 1.39–1.44 (m, 2H), 1.54–1.57 (m, 3H), 1.73–1.76 (m, 1H), 2.64–2.71 (m, 1H), 3.05–3.10 (m, 2H), 5.18 (d, *J* = 4.8 Hz, 1H), 6.08 (s, 2H), 6.101–6.105 (m, 2H), 7.19 (s, 1H), 7.49 (s, 1H), 7.74 (s, 1H), 7.76 (s, 1H), 7.84 (s, 1H); Anal. Calcd for C_22_H_21_NO_5_: C, 69.64; H, 5.58; N, 3.69. Found: C, 69.90; H, 5.75; N, 3.89.

*(2,3-Dimethoxy-6,7-methylenedioxyphenanthren-9-yl)(piperidin-2-yl)methanol (****27b****)*. Yield 62%; white powder; mp 245–247 °C; ^1^H NMR (400 MHz, DMSO-*d*_6_) *δ* 1.13–1.21 (m, 1H), 1.34–1.52 (m, 4H), 1.67–1.70 (m, 1H), 2.59–2.66 (m, 1H), 2.95–3.04 (m, 2H), 3.90 (s, 3H), 3.99 (s, 3H), 5.16 (br, 1H), 5.59 (br, 1H), 6.18 (s, 1H), 6.20 (s, 1H), 7.40 (s, 1H), 7.61 (s, 1H), 7.74 (s, 1H), 7.98 (s, 1H), 8.33 (s, 1H); Anal. Calcd for C_23_H_25_NO_5_: C, 69.86; H, 6.37; N, 3.54. Found: C, 69.13; H, 6.62; N, 3.79.

*(2,3-Methylenedioxy-6,7-dimethoxyphenanthren-9-yl)(piperidin-2-yl)methanol (****27c****)*. Yield 64%; white powder; mp 210–211 °C; ^1^H NMR (400 MHz, DMSO-*d*_6_) *δ* 1.15–1.31 (m, 3H), 1.43–1.46 (m, 1H), 1.54–1.57 (m, 1H), 1.70–1.73 (m, 1H), 2.42–2.48 (m, 1H), 2.86–2.94 (m, 2H), 3.91 (s, 3H), 4.01 (s, 3H), 5.06 (br, 1H), 5.3 (d, *J* = 2.8 Hz, 1H), 6.16 (s, 2H), 7.39 (s, 1H), 7.58 (s, 1H), 7.67 (s, 1H), 8.03 (s, 1H), 8.27 (s, 1H); ^13^C NMR (100 MHz, DMSO-*d*_6_) *δ* 24.35, 26.36, 27.09, 46.68, 55.48, 55.77, 60.74, 73.95, 100.89, 101.09, 104.26, 105.10, 105.30, 122.84, 123.97, 125.03, 125.18, 126.65, 134.36, 146.53, 147.48, 148.10, 148.68; Anal. Calcd for C_23_H_25_NO_5_: C, 69.86; H, 6.37; N, 3.54. Found: C, 69.15; H, 6.60; N, 3.81.

*(2,3-Ethylenedioxy-6,7-ethylenedioxyphenanthren-9-yl)(piperidin-2-yl)methanol (****27d****)*. Yield 74%; white powder; mp 250–252 °C; ^1^H NMR (400 MHz, DMSO-*d*_6_) *δ* 1.12–1.18 (m, 1H), 1.20–1.29 (m, 2H), 1.42–1.51 (m, 2H), 1.68–1.71 (m, 1H), 2.40–2.45 (m, 1H), 2.76–2.79 (m, 1H), 2.89–2.92 (m, 1H), 4.35 (s, 4H), 4.37 (s, 3H), 4.90–4.92 (m, 1H), 5.27 (d, *J* = 3.2 Hz, 1H), 7.32 (s, 1H), 7.53 (s, 1H), 7.58 (s, 1H), 8.00 (s, 1H), 8.05 (s, 1H); ^13^C NMR (100 MHz, DMSO-*d*_6_) *δ* 24.25, 26.29, 26.75, 46.56, 60.35, 64.22, 73.96, 109.13, 109.63,110.71, 113.84, 122.41, 124.06, 124.28, 125.09, 125.75, 134.07, 142.70, 143.16, 143.22, 143.50; HRMS (ESI) calcd for C_24_H_26_NO_5_ (M+H)^+^ 408.1805, found 408.1809.

*(6,7-Ethylenedioxy-2,3-dimethoxyphenanthren-9-yl)(piperidin-2-yl)methanol (****27e****)*. Yield 81%; white powder; mp 232–234 °C; ^1^H NMR (400 MHz, DMSO-*d*_6_) *δ*1.24–1.26 (m, 2H), 1.59–1.68 (m, 4H), 3.02–3.05 (m, 1H), 3.26–3.29 (m, 1H), 3.90 (s, 3H), 3.99 (s, 3H), 4.40 (s, 4H), 5.58 (br, 1H), 6.27 (d, *J* = 3.6 Hz, 1H), 7.43 (s, 1H), 7.62 (s, 1H), 7.77 (s, 1H), 7.96 (s, 1H), 8.28 (s, 1H), 8.31 (br, 1H), 9.30 (br, 1H); ^13^C NMR (100 MHz, DMSO-*d*_6_) *δ* 21.05, 21.28, 21.79, 44.09, 55.47, 55.81, 58.49, 64.24, 67.88, 103.70, 108.77, 109.71, 109.93, 122.64, 123.40, 123.63, 124.95, 125.19, 131.27, 143.23, 143.41, 148.77, 149.19; HRMS (ESI) calcd for C_24_H_28_NO_5_ (M+H)^+^ 410.1962, found 410.1963.

*2-[(2,3-Methylenedioxy-6,7-methylenedioxyphenanthren-9-yl)methyl]piperidine (****28a****)*. Yield 73%; white powder; mp 206–208 °C; ^1^H NMR (400 MHz, DMSO-*d*_6_) *δ* 1.15–1.24 (m, 3H), 1.33–1.38 (m, 1H), 1.48–1.51 (m, 1H), 1.58–1.60 (m, 1H), 1.67 (br, 1H), 2.77–2.78 (m, 1H), 2.89–2.98 (m, 2H), 3.02–307 (m, 1H), 6.15 (s, 2H), 6.19 (s, 2H), 7.31 (s, 1H), 7.43 (s, 1H), 7.57 (s, 1H), 8.16 (s, 1H), 8.21 (s, 1H); ^13^C NMR (100 MHz, CDCl_3_) *δ* 24.66, 25.77, 32.95, 41.54, 46.94, 56.40, 100.29, 100.96, 101.18, 101.35, 102.14, 105.13, 125.73, 126.24, 126.80, 127.02, 127.30, 130.56, 147.06, 147.19, 147.55, 147.75; Anal. Calcd for C_22_H_21_NO_4_: C, 72.71; H, 5.82; N, 3.85. Found: C, 72.98; H, 5.95; N, 4.01.

*2-[(2,3-Dimethoxy-6,7-methylenedioxyphenanthren-9-yl)methyl]piperidine (****28b****)*. Yield 82%; white powder; mp 172–174 °C; ^1^H NMR (400 MHz, CDCl_3_) *δ* 1.30–1.37 (m, 2H), 1.57–1.64 (m, 2H), 1.80–1.82 (m, 2H), 2.43–2.49 (m, 1H), 2.85–2.94 (m, 2H), 2.97–3.00 (m, 1H), 3.17–3.22 (m, 1H), 4.02 (s, 3H), 4.09 (s, 3H), 6.12 (s, 2H), 7.14 (s, 1H), 7.43 (s, 1H), 7.47 (s, 1H), 7.74 (s, 1H), 7.91 (s, 1H); Anal. Calcd for C_23_H_25_NO_4_: C, 72.80; H, 6.64; N, 3.69. Found: C, 72.97; H, 6.85; N, 3.88.

*2-[(2,3-Methylenedioxy-6,7-dimethoxyphenanthren-9-yl)methyl]piperidine (****28c****)*. Yield 85%; white powder; mp 148–150 °C; ^1^H NMR (400 MHz, CDCl_3_) *δ* 1.30–1.38 (m, 2H), 1.46–1.50 (m ,1H), 1.56–1.60 (m, 1H), 1.78–1.83 (m, 2H), 2.44–2.51 (m, 1H), 2.90–3.00 (m, 3H), 3.20–3.26 (m, 1H), 4.05 (s, 3H), 4.10 (s, 3H), 6.08 (s, 2H), 7.14 (s, 1H), 7.39 (s, 1H), 7.44 (s, 1H), 7.80 (s, 1H), 7.84 (s, 1H); ^13^C NMR (100 MHz, CDCl_3_) *δ* 25.02, 26.23, 33.60, 41.72, 47.21, 55.89, 55.92, 56.47, 100.10, 101.14, 103.41, 105.28, 125.21, 125.51, 125.54, 125.79, 127.28, 130.58, 146.89, 147.60, 148.55, 148.88; Anal. Calcd for C_23_H_25_NO_4_: C, 72.80; H, 6.64; N, 3.69. Found: C, 72.91; H, 6.80; N, 3.79.

*2-[(2,3-Ethylenedioxy-6,7-ethylenedioxyphenanthren-9-yl)methyl]piperidine (****28d****)*. Yield 80%; white powder; mp 110–112 °C; ^1^H NMR (400 MHz, DMSO-*d*_6_) *δ* 1.07–1.21 (m, 2H), 1.26–1.37 (m, 1H), 1.44–1.47 (m, 1H), 1.54–1.56 (m, 1H), 1.65–1.68 (m, 1H), 2.39–2.45 (m, 1H), 2.71–2.72 (m, 1H), 2.81–2.96 (m, 3H), 4.35 (s, 4H), 4.38 (s, 4H), 7.25 (s, 1H), 7.27 (s, 1H), 7.49 (s, 1H), 7.99 (s, 1H), 8.05 (s, 1H); ^13^C NMR (100 MHz, CDCl_3_) *δ* 24.88, 26.09, 33.35, 41.66, 47.08, 56.05, 64.51, 64.57, 109.22, 109.89, 111.09, 113.84, 124.64, 125.43, 125.86, 126.09, 126.55, 130.41, 143.00, 143.32, 143.35, 143.41; HRMS (ESI) calcd for C_24_H_26_NO_4_ (M+H)^+^ 392.1856, found 392.1856.

*2-[(6,7-Ethylenedioxy-2,3-dimethoxyphenanthren-9-yl)methyl]piperidine (****28e****)*. Yield 92%; white powder; mp 173–176 °C; ^1^H NMR (400 MHz, DMSO-*d*_6_) *δ* 1.24–1.30 (m, 1H), 1.34–1.44 (m, 1H), 1.51–1.54 (m, 1H), 1.62–1.70 (m, 3H), 2.75–2.81 (m, 1H), 2.97–3.02 (m, 1H), 3.19–3.21 (m, 2H), 3.28–3.34 (m, 1H), 3.89 (s, 3H), 3.98 (s, 3H), 4.40 (s, 4H), 7.31 (s, 1H), 7.43 (s, 1H), 7.60 (s, 1H), 7.95 (s, 1H), 8.25 (s, 1H); HRMS (ESI) calcd for C_24_H_28_NO_4_ (M+H)^+^ 394.2013, found 394.2016.

*2,3-Methylenedioxy-6,7-methylenedioxyphenanthro[9,10-b]quinolizidine (****10****)*. Yield 60%; white powder; mp 289–291 °C; ^1^H NMR (400 MHz, CDCl_3_) *δ* 1.41–1.55 (m, 2H), 1.74–1.80 (m, 2H), 1.86–1.89 (m, 1H), 2.00–2.03 (m, 1H), 2.25–2.38 (m, 2H), 2.82–2.89 (m, 1H), 3.03–3.08 (m, 1H), 3.24–3.27 (m, 1H), 3.55 (d, *J* = 15.6 Hz, 1H), 4.30 (d, *J* = 15.6 Hz, 1H), 6.07–6.08 (m, 4H), 7.19 (s, 1H), 7.30 (s, 1H), 7.79 (s, 1H), 7.80 (s, 1H); ^13^C NMR (100 MHz, CDCl_3_) *δ* 24.32, 25.95, 33.75, 35.11, 55.25, 56.45, 57.49, 100.29, 100.76, 100.90, 101.13, 101.21, 125.18, 125.37, 125.53, 125.75, 126.59, 147.01, 147.14, 147.22; HRMS (ESI) calcd for C_23_H_22_NO_4_ (M+H)^+^ 376.1543, found 376.1545.

*2,3-Methylenedioxy-6,7-dimethoxyphenanthro[9,10-b]quinolizidine (****11****)*. Yield 90%; white powder; mp 239–241 °C; ^1^H NMR (400 MHz, CDCl_3_) *δ* 1.42–1.57 (m, 2H), 1.72–1.90 (m, 3H), 2.00–2.03 (m, 1H), 2.27–2.39 (m, 2H), 2.84–2.91 (m, 1H), 3.05–3.10 (m, 1H), 3.27–3.30 (m, 1H), 3.59 (d, *J* = 15.2 Hz, 1H), 4.04 (s, 3H), 4.08 (s, 3H), 4.09 (d, *J* = 15.2 Hz, 1H), 6.08–6.09 (m, 2H), 7.11 (s, 1H), 7.32 (s, 1H), 7.78 (s, 1H), 7.86 (s, 1H); ^13^C NMR (100 MHz, CDCl_3_) *δ* 24.30, 25.95, 33.71, 35.04, 55.81, 55.89, 56.16, 56.30, 57.39, 100.45, 101.17, 101.21, 102.69, 103.36, 123.65, 123.78, 124.83, 125.00, 125.49, 126.42, 146.95, 146.97, 148.22, 148.61; HRMS (ESI) calcd for C_24_H_26_NO_4_ (M+H)^+^ 392.1856, found 392.1859.

*2,3-Dimethoxy-6,7-methylenedioxyphenanthro[9,10-b]quinolizidine (****12****)*. Yield 87%; white powder; mp 276–278 °C; ^1^H NMR (400 MHz, CDCl_3_) *δ* 1.42–1.58 (m, 2H), 1.75–1.80 (m, 2H), 1.87–1.90 (m, 1H), 2.02–2.06 (m, 1H), 2.26–2.33 (m, 1H), 2.36–2.42 (m, 1H), 2.85–2.92 (m, 1H), 3.09–3.14 (m, 1H), 3.25–3.30 (m, 1H), 3.57 (d, *J* = 15.2 Hz, 1H), 4.05 (s, 3H), 4.09 (s, 3H), 4.33 (d, *J* = 15.2 Hz, 1H), 6.08 (s, 2H), 7.22 (s, 1H), 7.23 (s, 1H), 7.78 (s, 1H), 7.88(s, 1H); ^13^C NMR (100 MHz, CDCl_3_) *δ* 24.33, 25.93, 33.77, 34.78, 55.86, 55.88, 56.23, 56.42, 57.40, 100.42, 100.64, 101.20, 103.25, 103.54, 123.84, 124.69, 125.02, 125.15, 125.27, 146.88, 147.02, 148.39, 148.66; HRMS (ESI) calcd for C_24_H_26_NO_4_ (M+H)^+^ 392.1856, found 392.1854.

*2,3-Ethylenedioxy-6,7-ethylenedioxyphenanthro[9,10-b]quinolizidine (****13****)*. Yield 51%; white powder; mp 243–245 °C; ^1^H NMR (400 MHz, CDCl_3_) *δ* 1.44–1.54 (m, 2H), 1.79–1.88 (m, 3H), 1.98–2.01 (m, 1H), 2.29–2.36 (m, 2H), 2.79–2.86 (m, 1H), 3.00–3.04 (m, 1H), 3.24–3.26 (m, 1H), 3.49–3.53 (m, 1H), 4.29 (d, *J* = 16.0 Hz, 1H), 4.37 (s, 8H), 7.22 (s, 1H), 7.33 (s, 1H), 7.86 (s, 1H), 7.87 (s, 1H); ^13^C NMR (100 MHz, CDCl_3_) *δ* 24.26, 25.84, 33.58, 34.54, 55.97, 56.20, 57.52, 64.59, 109.13, 109.63, 109.76, 110.04, 124.27, 124.41, 124.62, 125.18, 125.97, 142.80, 142.94, 143.13, 143.15; HRMS (ESI) calcd for C_25_H_26_NO_4_ (M+H)^+^ 404.1856, found 404.1855.

*2,3-Ethylenedioxy-6,7-dimethoxyphenanthro[9,10-b]quinolizidine (****14****)*. Yield 48%; white powder; mp 256–258 °C; ^1^H NMR (400 MHz, CDCl_3_) *δ* 1.46–1.55 (m, 2H), 1.81–1.89 (m, 3H), 2.00–2.03 (m, 1H), 2.32–2.38 (m, 2H), 2.84–2.90 (m, 1H), 3.05–3.10 (m, 1H), 3.28–3.31 (m, 1H), 3.56–3.60 (m, 1H), 4.04 (s, 3H), 4.07 (s, 3H), 4.35 (d, *J* = 16.0 Hz, 1H), 4.39 (s, 4H), 7.10 (s, 1H), 7.38 (s, 1H), 7.80 (s, 1H), 7.93 (s, 1H); ^13^C NMR (100 MHz, CDCl_3_) *δ* 24.28, 25.89, 33.63, 34.64, 55.87, 55.97, 56.10, 56.32, 57.49, 64.61, 64.65, 103.07, 103.65, 109.18, 109.98, 123.30, 123.82, 124.44, 125.08, 125.95, 142.94, 143.04, 148.26, 148.67; HRMS (ESI) calcd for C_25_H_28_NO_4_ (M+H)^+^ 406.2013, found 406.2014.

*2-[(2,3-Ethylenedioxy-6,7-dimethoxyphenanthren-9-yl)methyl]pyridine (****29****).* White powder; mp 193–194 °C; ^1^H NMR (400 MHz, CDCl_3_) *δ* 3.85 (s, 3H), 4.04 (s, 3H), 4.37 (s, 4H), 4.56 (s, 2H), 7.01–7.02 (m, 1H), 7.06–7.09 (m, 1H), 7.28 (s, 1H), 7.36 (s, 1H), 7.42–7.46 (m, 2H), 7.78 (s, 1H), 7.91 (s, 1H), 8.58 (d, *J* = 4.8 Hz, 1H); ^13^C NMR (100 MHz, CDCl_3_) *δ* 43.69, 55.79, 55.84, 64.53, 64.63, 103.41, 106.16, 108.82, 113.97, 121.24, 122.91, 124.99, 125.01, 125.42, 125.53, 126.85, 131.52, 136.49, 143.23, 143.55, 148.48, 148.67, 148.97, 161.09; Anal. Calcd for C_24_H_21_NO_4_: C, 74.40; H, 5.46; N, 3.62. Found: C, 74.65; H, 5.71; N, 3.86.

*2-[(2,3-Ethylenedioxy-6,7-dimethoxyphenanthren-9-yl)methyl]piperidine (****30****).* White powder; mp 173–175 °C; ^1^H NMR (400 MHz, CDCl_3_) *δ* 1.26–1.31 (m, 1H), 1.36–1.42 (m, 1H), 1.53–1.62 (m, 2H), 1.76–1.83 (m, 2H), 2.50–2.56 (m, 1H), 2.95–2.96 (m, 1H), 3.05–3.08 (m, 2H), 3.18–3.23 (m, 1H), 4.06 (s, 3H), 4.08 (s, 3H), 4.38 (s, 4H), 7.24 (s, 1H), 7.38 (s, 1H), 7.40 (s, 1H), 7.81 (s, 1H), 7.90 (s, 1H); ^13^C NMR (100 MHz, CDCl_3_) *δ* 24.69, 25.75, 32.94, 41.20, 46.88, 55.87, 56.06, 56.36, 64.50, 64.60, 103.63, 105.15, 108.71, 113.77, 124.69, 124.98, 125.16, 125.32, 126.63, 130.30, 143.24, 143.44, 148.56, 148.79; Anal. Calcd for C_24_H_27_NO_4_: C, 73.26; H, 6.92; N, 3.56. Found: C, 73.51; H, 7.18; N, 3.73.

*6,7-Ethylenedioxy-2,3-dimethoxyphenanthro[9,10-b]quinolizidine (****15****).* White powder; mp 248–249 °C; ^1^H NMR (400 MHz, CDCl_3_) *δ* 1.45–1.56 (m, 2H), 1.80–1.90 (m, 3H), 2.01–2.04 (m, 1H), 2.30–2.38 (m, 2H), 2.84–2.90 (m, 1H), 3.05–3.10 (m, 1H), 3.25–3.28 (m, 1H), 3.53–3.57 (m, 1H), 4.04 (s, 3H), 4.07 (s, 3H), 4.33 (d, *J* = 16.0 Hz, 1H), 4.39 (s, 4H), 7.21 (s, 1H), 7.27 (s, 1H), 7.79 (s, 1H), 7.94 (s, 1H); ^13^C NMR (100 MHz, CDCl_3_) *δ* 24.35, 25.94, 33.76, 34.73, 55.88, 55.90, 56.11, 56.21, 57.47, 64.63, 103.52, 103.89, 109.07, 109.27, 123.41, 124.28, 124.61, 124.78, 125.17, 125.29, 142.78, 143.00, 148.34, 148.64; HRMS (ESI) calcd for C_25_H_28_NO_4_ (M+H)^+^ 406.2013, found 406.2011.

*(E)-Methyl 2-(3,4-dimethoxyphenyl)-3-(4-hydroxyphenyl)acrylate (****31****).* White powder; mp 183–184 °C; ^1^H NMR (400 MHz, CDCl_3_) *δ* 3.66 (s, 3H), 3.68 (s, 3H), 3.79 (s, 3H), 6.60–6.62 (m, 2H), 6.68–6.71 (m, 1H), 6.75 (s, 1H), 6.95–6.98 (m, 3H), 7.67 (s, 1H), 9.91 (s, 1H); ^13^C NMR (100 MHz, CDCl_3_) *δ* 51.84, 55.30, 55.43, 111.87, 113.09, 115.23, 121.77, 125.18, 128.36, 128.46, 132.37, 139.69, 148.28, 148.83, 158.74, 167.81; Anal. Calcd for C_18_H_18_O_5_: C, 68.78; H, 5.77. Found: C, 68.94; H, 5.89.

*Methyl 3-hydroxy-6,7-dimethoxyphenanthrene-9-carboxylate (****32****).* Yellow powder; mp 198–199 °C；^1^H NMR (400 MHz, CDCl_3_) *δ* 3.91 (s, 3H), 3.94 (s, 3H), 4.03 (s, 3H), 7.17 (dd, *J* = 8.8 Hz, *J* = 2.0 Hz, 1H), 7.94–7.98 (m, 3H), 8.41 (s, 1H), 8.47 (s, 1H), 10.20 (s, 1H); ^13^C NMR (100 MHz, CDCl_3_) *δ* 51.88, 55.16, 55.41, 103.74, 106.03, 106.35, 117.29, 120.20, 122.62, 124.00, 124.30, 130.82, 131.90, 133.30, 148.64, 149.42, 158.60, 167.47; Anal. Calcd for C_18_H_16_O_5_: C, 69.22; H, 5.16. Found: C, 69.49; H, 5.38.

*Methyl 3-(benzyloxy)-6,7-dimethoxyphenanthrene-9-carboxylate (****33****).* White powder; mp 136–138 °C; ^1^H NMR (400 MHz, CDCl_3_) *δ* 4.02 (s, 3H), 4.08 (s, 3H), 4.10 (s, 3H), 5.31 (s, 2H), 7.29 (d, *J* = 9.2 Hz, 1H), 7.35–7.39 (m, 1H), 7.42–7.45 (m, 2H), 7.54–7.55 (m, 2H), 7.87 (d, *J* = 8.8 Hz, 1H), 7.81 (s, 1H), 7.91 (s, 1H), 8.45 (s, 1H)，8.65 (s, 1H); ^13^C NMR (100 MHz, CDCl_3_) *δ* 51.86, 55.61, 55.70, 70.18, 102.83, 104.92, 106.68, 116.14, 121.32, 124.09, 124.73, 124.88, 127.50, 128.13, 128.66, 131.09, 131.61, 133.12, 136.71, 148.54, 149.61, 159.09, 167.96; Anal. Calcd for C_25_H_22_O_5_: C, 74.61; H, 5.51. Found: C, 74.49; H, 5.39.

*3-(Benzyloxy)-6,7-dimethoxyphenanthrene-9-carboxylic acid (****34****).* White powder; mp 222–224 °C; ^1^H NMR (400 MHz, DMSO-*d*_6_) *δ* 3.93 (s, 3H), 4.07 (s, 3H), 5.41 (s, 2H), 7.36 (t, *J* = 7.2 Hz, 2H), 7.44 (t, *J* = 7.4 Hz, 2H), 7.59 (d, *J* = 7.5 Hz, 2H), 8.04 (d, *J* = 8.8 Hz, 1H), 8.10 (s, 1H), 8.21 (s, 1H), 8.46 (s, 1H), 8.59 (s, 1H), 12.97 (br, 1H); ^13^C NMR (100 MHz, DMSO-*d*_6_) *δ* 55.20, 55.78, 69.72 104.17, 105.29, 106.70, 116.94, 122.41, 123.78, 124.38, 124.65, 128.01, 128.08, 128.51, 130.27, 131.71, 136.90, 148.79, 149.44, 158.94, 168.90; Anal. Calcd for C_24_H_20_O_5_: C, 74.21; H, 5.19. Found: C, 74.47; H, 5.36.

*(3-(Benzyloxy)-6,7-dimethoxyphenanthren-9-yl)(pyridin-2-yl)methanone (****35****).* Yellow powder; mp 202–204 °C; ^1^H NMR (400 MHz, CDCl_3_) *δ* 3.78 (s, 3H), 4.07 (s, 3H), 5.42 (s, 2H), 7.32–7.39 (m, 2H), 7.43–7.46 (m, 2H), 7.59–7.61 (m, 2H), 7.67–7.70 (m, 1H), 7.76 (s, 1H), 7.92–7.95 (m, 2H), 8.11–8.15 (m, 3H), 8.25 (s, 1H), 8.68 (d, *J* = 4.8 Hz, 1H); ^13^C NMR (100 MHz, CDCl_3_) *δ* 55.10, 55.86, 69.77, 104.29, 105.51, 106.35, 116.82, 123.52, 124.16, 124.27, 124.51, 126.79, 128.00, 128.12, 128.49, 129.50, 131.23, 131.74, 132.40, 136.90, 137.50, 148.88, 149.03, 149.49, 155.70, 159.00, 196.26; Anal. Calcd for C_29_H_23_NO_4_: C, 77.49; H, 5.16; N, 3.12. Found: C, 77.71; H, 5.44; N, 3.36.

*(3-Hydroxy-6,7-dimethoxyphenanthren-9-yl)(pyridin-2-yl)methanol (****36****).* White powder; mp 230–231 °C; ^1^H NMR (400 MHz, DMSO-*d*_6_) *δ* 3.80 (s, 3H), 3.97 (s, 3H), 6.27 (m, 2H), 7.10 (d, *J* = 8.4 Hz, 1H), 7.21–7.24 (m, 1H), 7.57–7.59 (m, 1H), 7.70–7.82 (m, 4H), 7.90 (m, 2H), 8.47(d, *J* = 4.0 Hz, 1H), 9.73 (s, 1H); ^13^C NMR (100 MHz, DMSO-*d*_6_) *δ* 55.25, 55.44, 74.65, 103.91, 105.93, 106.20, 116.63, 121.12, 122.89, 124.19, 124.74, 130.16, 130.74, 133.92, 136.65, 148.07, 148.15, 148.39, 156.07, 163.76; Anal. Calcd for C_22_H_19_NO_4_: C, 73.12; H, 5.30; N, 3.88. Found: C, 73.39; H, 5.55; N, 3.99.

*2-[(3-Hydroxy-6,7-dimethoxyphenanthren-9-yl)methyl]pyridine (****37****).* White powder; mp 235–236 °C; ^1^H NMR (400 MHz, CDCl_3_) *δ* 3.80 (s, 3H), 3.98 (s, 3H), 4.51 (s, 2H), 7.09–7.11 (m, 1H), 7.17–7.20 (m, 1H), 7.25–7.27 (m, 1H), 7.54 (s, 1H), 7.59 (s, 1H), 7.62–7.66 (m, 1H), 7.72–7.74 (m, 1H), 7.92 (s, 2H), 8.51 (d, *J* = 4.4 Hz, 1H), 9.71 (s, 1H); ^13^C NMR (100 MHz, CDCl_3_) *δ* 42.43, 55.24, 55.49, 104.05, 105.97, 106.06, 116.60, 121.30, 122.95, 124.20, 124.52, 125.75, 126.04, 129.58, 130.02, 130.58, 136.48, 148.34, 148.74, 148.79, 155.89, 160.68; Anal. Calcd for C_22_H_19_NO_3_: C, 76.50; H, 5.54; N, 4.06. Found: C, 76.79; H, 5.75; N, 4.31.

*2-[(3-Hydroxy-6,7-dimethoxyphenanthren-9-yl)methyl]piperidine (****38****).* White powder; mp 142–144 °C; ^1^H NMR (400 MHz, DMSO-*d*_6_) *δ* 1.14–1.24 (m, 2H), 1.27–1.36 (m, 1H), 1.45–1.48 (m, 1H), 1.57–1.60 (m, 1H), 1.68 (br, 1H), 2.42–2.47 (m, 1H), 2.78–2.80 (m, 1H), 2.92–3.06 (m, 3H), 3.95 (s, 3H), 4.01 (s, 3H), 7.08 (d, *J* = 8.8 Hz, 1H), 7.41 (s, 1H), 7.45 (s, 1H), 7.69–7.71 (m, 1H), 7.92 (s, 1H), 7.95 (s, 1H), 9.71 (br, 1H); ^13^C NMR (100 MHz, DMSO-*d*_6_) *δ* 24.51, 25.92, 32.47, 40.67, 46.57, 55.50, 56.61, 104.17, 105.37, 105.91, 116.67, 124.14, 124.34, 125.45, 126.28, 128.99, 129.37, 130.37, 148.44, 148.92, 155.91; HRMS (ESI) calcd for C_22_H_26_NO_3_ (M+H)^+^ 352.1907, found 352.1912.

*2-[(3-(Benzyloxy)-6,7-dimethoxyphenanthren-9-yl)methyl]piperidine (****39****).* White powder; mp 70–72 °C。^1^H NMR (400 MHz, DMSO-*d*_6_) *δ* 1.13–1.21 (m, 2H), 1.29–1.32 (m, 1H), 1.44–1.47 (m, 1H), 1.56–1.59 (m, 1H), 1.67–1.70 (m, 1H), 2.40–2.46 (m, 2H), 2.78–2.80 (m, 1H), 2.90–2.93 (m, 1H), 2.98–3.03 (m, 1H), 3.95 (s, 3H), 4.04 (s, 3H), 5.36 (s, 2H), 7.26 (dd, *J* = 2.4 Hz, *J* = 8.8 Hz, 1H), 7.34–7.37 (m, 1H), 7.41–7.46 (m, 4H), 7.58–7.59 (m, 2H), 7.79 (d, *J* = 8.8 Hz, 1H), 8.07 (s, 1H), 8.15 (d, *J* = 2.0 Hz, 1H); ^13^C NMR (100 MHz, CDCl_3_) *δ* 24.76, 25.84, 33.10, 41.29, 46.98, 55.99, 56.10, 56.47, 70.58, 103.91, 105.07, 105.65, 115.95, 124.97, 125.93, 126.64, 127.61, 128.12, 128.72, 129.75, 130.43, 137.14, 148.76, 149.28, 157.15; HRMS (ESI) calcd for C_29_H_32_NO_3_ (M+H)^+^ 442.2377, found 442.2382.

*6-Benzyloxy-2,3-dimethoxyphenanthro[9,10-b]quinolizidine (****40****).* White powder; mp 222–224 °C; ^1^H NMR (400 MHz, CDCl_3_) *δ* 1.43–1.46 (m, 1H), 1.50–1.59 (m, 1H), 1.77–1.81 (m, 2H), 1.87–1.90 (m, 1H), 2.02–2.05 (m, 1H), 2.31–2.34 (m, 1H), 2.85–2.92 (m, 1H), 3.06–3.10 (m, 1H), 3.26–3.29 (m, 1H), 3.61–3.65 (m, 1H), 4.05 (s, 3H), 4.08 (s, 3H), 4.44 (d, *J* = 15.2 Hz, 1H), 5.27 (s, 2H), 7.24–7.26 (m, 2H), 7.34–7.37 (m, 1H), 7.40–7.44 (m, 2H), 7.53–7.55 (m, 2H), 7.79 (d, *J* = 11.2 Hz, 1H), 7.82 (s, 1H), 7.97 (s, 1H); ^13^C NMR (100 MHz, CDCl_3_) *δ* 24.32, 25.90, 33.73, 34.68, 55.94, 55.96, 56.06, 56.25, 57.59, 70.51, 103.81, 106.35, 115.34, 123.42, 123.80, 124.08, 124.56, 126.42, 127.64, 128.12, 128.72, 130.09, 137.18, 148.30, 149.36, 156.64; HRMS (ESI) calcd for C_30_H_32_NO_3_ (M+H)^+^ 454.2377, found 454.2380.

*6-Hydroxy-2,3-dimethoxyphenanthro[9,10-b]quinolizidine (****16****).* Light pink powder; mp 188–190 °C; ^1^H NMR (400 MHz, DMSO-*d*_6_) *δ* 1.32–1.40 (m, 2H), 1.57–1.60 (m, 1H), 1.69–1.79 (m, 2H), 1.93–1.95 (m, 1H), 2.11–2.23 (m, 2H), 2.66–2.72 (m, 1H), 3.04–3.08 (m, 1H), 3.39–3.43 (m, 1H),3.92 (s, 3H), 3.98 (s, 3H), 4.10–4.11 (m, 1H), 4.30 (d, *J* = 15.6 Hz, 1H), 7.07–7.09 (m, 1H), 7.24 (s, 1H), 7.70–7.72 (m, 1H), 7.91 (s, 1H), 7.94 (s, 1H), 9.65 (s, 1H); ^13^C NMR (100 MHz, DMSO-*d*_6_) *δ* 24.01，25.46, 33.13, 33.90, 55.37, 55.46, 55.52, 57.24, 103.95, 103.99, 106.52, 116.22, 121.88, 122.57, 123.37, 123.95, 125.40, 125.62, 129.90, 148.04, 149.05, 155.26; HRMS (ESI) calcd for C_23_H_26_NO_3_ (M+H)^+^ 364.1907, found 364.1911.

*2-[(3-Cyclopropylmethoxy-6,7-dimethoxyphenanthren-9-yl)methyl]pyridine (****41****).* White powder; mp 140–141 °C; ^1^H NMR (400 MHz, CDCl_3_) *δ* 0.44–0.45 (m, 2H), 0.71–0.74 (m, 2H), 1.34–1.44 (m, 1H), 3.86 (s, 3H), 4.02 (d, *J* = 6.8 Hz, 2H), 4.08 (s, 3H), 4.60 (s, 2H), 7.04–7.10 (m, 2H), 7.20–7.22 (m, 1H), 7.41 (s, 1H), 7.44–7.48 (m, 1H), 7.58 (s, 1H), 7.77–7.79 (m, 1H), 7.89 (br, 2H), 8.59 (d, *J* = 4.4 Hz, 1H); ^13^C NMR (100 MHz, CDCl_3_) *δ* 3.29, 10.45, 43.66, 55.79, 55.97, 73.09, 103.73, 105.30, 106.10, 115.46, 121.22, 122.93, 124.94, 126.01, 126.25, 126.71, 129.77, 130.61, 130.80, 136.45, 148.57, 148.98, 149.13, 157.45, 161.12; Anal. Calcd for C_26_H_25_NO_3_: C, 78.17; H, 6.31; N, 3.51. Found: C, 78.44; H, 6.42; N, 3.75.

*2-[(3-Cyclopropylmethoxy-6,7-dimethoxyphenanthren-9-yl)methyl]piperidine (****42****).* White powder; mp 145–147 °C; ^1^H NMR (400 MHz, CDCl_3_) *δ* 0.42–0.46 (m, 2H), 0.69–0.74 (m, 2H), 1.30–1.41 (m, 3H), 1.43–1.52 (m, 1H), 1.56–1.59 (m, 1H), 1.79–1.83 (m, 2H), 2.45–2.50 (m, 1H), 2.92–3.00 (m, 3H), 3.21–3.24 (m, 1H), 4.01 (d, *J* = 6.8 Hz, 1H), 4.06 (s, 3H), 4.11 (s, 3H), 7.19 (d, *J* = 8.4 Hz, 1H), 7.41 (s, 1H), 7.50 (s, 1H), 7.72–7.74 (m, 1H), 7.87 (s, 1H), 7.93 (s, 1H); ^13^C NMR (100 MHz, CDCl_3_) *δ* 3.30, 10.47, 25.04, 26.26, 33.62, 41.72, 47.22, 55.92, 56.04, 56.43, 73.13, 104.01, 105.11, 105.30, 115.55, 124.99, 125.82, 125.87, 126.72, 129.66, 129.78, 130.47, 148.71, 149.17, 157.34; HRMS (ESI) calcd for C_26_H_32_NO_3_ (M+H)^+^ 406.2377, found 406.2379.

*6-Cyclopropylmethoxy-2,3-dimethoxyphenanthro[9,10-b]quinolizidine (****17****).* White powder (0.87 g, 80%); mp 231–233 °C; ^1^H NMR (400 MHz, CDCl_3_) *δ* 0.42–0.46 (m, 2H), 0.70–0.74 (m, 2H), 1.34–1.42 (m, 1H), 1.43–1.59 (m, 2H), 1.82–1.91 (m, 3H), 2.03–2.06 (m, 1H), 2.29–2.42 (m, 2H), 2.88–2.94 (m, 1H), 3.08–3.14 (m, 1H), 3.28–3.31 (m, 1H), 3.64–3.67 (m, 1H), 4.01 (d, *J* = 7.2 Hz, 1H), 4.06 (s, 3H), 4.10 (s, 3H), 4.46 (d, *J* = 15.2 Hz, 1H), 7.20 (dd, *J* = 2.4 Hz, *J* = 9.2 Hz, 1H), 7.25 (s, 1H), 7.77–7.79 (m, 1H), 7.89–7.93 (m, 2H); ^13^C NMR (100 MHz, CDCl_3_) *δ* 3.29, 10.46, 24.31, 25.91, 33.74, 34.66, 55.88, 56.00, 56.22, 57.50, 73.01, 103.76, 103.83, 105.98, 114.84, 123.37, 123.63, 123.99, 124.36, 126.40, 130.10, 148.21, 149.27, 156.75; HRMS (ESI) calcd for C_27_H_32_NO_3_ (M+H)^+^ 418.2377, found 418.2381.
